# Invasion of Epithelial Cells Is Correlated with Secretion of Biosurfactant via the Type 3 Secretion System (T3SS) of *Shigella flexneri*

**DOI:** 10.1155/2020/3062821

**Published:** 2020-07-27

**Authors:** Duchel Jeanedvi Kinouani Kinavouidi, Christian Aimé Kayath, Etienne Nguimbi

**Affiliations:** ^1^Laboratoire de Biologie Cellulaire et Moléculaire (BCM), Faculté des Sciences et Techniques, Université Marien N'gouabi, BP. 69, Brazzaville, Congo; ^2^Institut National de Recherche en Sciences Exactes et Naturelles (IRSEN), Avenue de l'Auberge Gascogne, B.P 2400, Brazzaville, Congo

## Abstract

Biosurfactants are amphipathic molecules produced by many microorganisms, usually bacteria, fungi, and yeasts. They possess the property of reducing the tension of the membrane interfaces. No studies have been conducted on *Shigella* species showing the role of biosurfactant-like molecules (BLM) in pathogenicity. The aim of this study is to assess the ability of *Shigella* environmental and clinical strains to produce BLM and investigate the involvement of biosurfactants in pathogenicity. Our study has shown that BLM are secreted in the extracellular medium with EI24 ranging from 80% to 100%. The secretion is depending on the type III secretion system (T3SS). Moreover, our results have shown that *S. flexneri*, *S. boydii,* and *S. sonnei* are able to interact with hydrophobic areas with 17.64%, 21.42%, and 22.22% hydrophobicity, respectively. BLM secretion is totally prevented due to inhibition of T3SS by 100 mM benzoic and 1.5 mg/ml salicylic acids. *P. aeruginosa* harboring T3SS is able to produce 100% of BLM in the presence or in the absence of both T3SS inhibitors. The secreted BLM are extractable with an organic solvent such as chloroform, and this could entirely be considered a lipopeptide or polypeptide compound. Secretion of BLM allows some *Shigella *strains to induce multicellular phenomena like “swarming.”

## 1. Introduction

The ingestion of pathogenic and virulent microorganisms generally affects peoples in both developed and developing countries. *Shigella* is one of the Gram-negative bacterium belonging to *Enterobacteriaceae* family and is a causative agent of bacillary dysentery or shigellosis [1]. Children under five years are the most affected. More and more shigellosis is considered like neglected disease; meanwhile, 164300 of death per years have been notified all over the world in 2010. Most deaths occur in sub-Saharan Africa and in south Asia [2, 3]. This includes Republic of Congo, and surprisingly, no epidemiological studies have been conducted in this field. The genus *Shigella* includes four species (*S. flexneri*, *S. sonnei*, *S. dysenteriae*, and *S. boydii*). Ten bacteria of *S. dysenteriae* type 1, and 100 to 180 bacteria of *S. flexneri* or *S. sonnei* are enough to produce symptomatic infection.

*Shigella*'s pathogenicity is based on a virulence plasmid pWR100 in which the mxi-spa locus encodes the type three secretion system (T3SS) involved in effector production like IpaB, C, and D (translocator and tip) to invade host cell [4]. A previous study in our laboratory showed that *Shigella* sp. isolated from Brazzaville wastewater were able to emulsify hydrocarbon from gasoline and/or diesel fuel [5]. According to amphipathic features, biosurfactants display a variety of surface activities, which explain their application in several fields related with emulsification, foaming, detergency, wetting, dispersion, pathogenicity, and solubilisation of hydrophobic compounds [6, 7]. Biosurfactants are produced from a couple of Gram negative bacteria like *Pseudomonas aeruginosa* and *Acinetobacter calcoaceticus*. Rhamnolipids are well known among biosurfactant, and many informations have been documented in terms of biochemical and biotechnological applications as well [8].

*Shigella* pathogenicity mechanisms have been mostly studied using S. flexneri 5a M90T strain as a Gram-negative bacterium model. In this way, this work aims to study the involvement of BLM via the Type Three Secretion System (T3SS) pathways. In addition, this work will assess the approvals that *Shigella* could use the BLM to promote the invasion and the dissemination inside epithelial cells.

## 2. Materials and Methods

### 2.1. Strains and Culture Conditions

Four *Shigella* strains were kindly provided and collected from laboratory of Molecular Bacteriology (Faculté de Médecine, Erasme Campus, Free University of Brussels). These included *S. flexneri*5a M90T, *S. flexneri spa40*-, *S. sonnei*, and *S. boydii*. Three pure culture strains were isolated from patients in Brazzaville University and Hospital Center (CHU-B) in 2018. These were provided by the Virology and Bacteriology Laboratory of this afore hospital. Thirty *Shigella* sp. strains were isolated in environmental wastewater of Brazzaville using decimal dilution in SS medium. Lab strains like *P. aeruginosa* and *E. coli* Top10 were used as controls in this study. The strains were spread on the plates containing LB medium with Congo red with 100 *μ*g/mL streptomycin for 24 hours at 37°C for wild type and 50 *μ*g/mL for *spa40* mutant.

### 2.2. Emulsification Index (EI24) Assay

From 5 mL of bacterial overnight culture, the emulsification index (EI24) was calculated as an indicator for BLM production as previously demonstrated [5]. The medium was adjusted to pH 7.2 and supplemented with gasoline or diesel fuel (1 mL for 300 mL of medium). The EI24 was investigated by adding fuels with LB medium in 1 : 1 ratio (v/v). The solution was vortexed for 5 min and incubated for 24 h at 37°C. The emulsification rate was calculated through the height of the emulsion layer. In addition, EI24 was determined for gasoline and diesel fuel hydrocarbons. All the experiments were performed in triplicate; EI24 = height of emulsion layer/total height of solution × 100.

### 2.3. Bacterial Swarming Assays

Swarming was studied for all *Shigella* strain used in this study, using plate assays containing 0.5% noble agar and LB medium with 0.5% dextrose. The mixture was sterilized at 121°C, during 15 min. After sterilization, the medium was supplemented with adequate antibiotics including streptomycin 100 *μ*g/mL for wild type and kanamycin 50 *μ*g/mL for the *Shigella flexneri* spa40 mutant. Approximately 6 h after pouring the plates, bacteria were inoculated and spread by using a sterilized platinum wire with log-phase cells ((OD600) 0.6) grown in their respective media used for the swarming experiments. Swarming plates that were imaged only for their comparative endpoint swarming development were incubated at 30°C for 24 h prior to imaging.

### 2.4. Bacterial Adhesion Assay

The adhesion of bacteria to hydrophobic surface was evaluated according to the method described by Rosenberg [9]. The hydrophobicity was evaluated according to the following formula: %H = *A*0 − *A*/*A*0 ∗ 100 with *A*_0_: OD before the mix and *A*: OD after vortexing of aqueous phase.

### 2.5. Induction Assay Using Congo Red

*Shigella* sp. have been cultivated in 5 mL of the final volume. One  mL of overnight culture was centrifuged and 500 *μ*L of sterile PBS and 10 *μ*l of Congo red (10 mg/ml) have been gently added and mixed with the pellet by avoiding breakage of the cells. Samples were incubated at 37°C with stirring. After 30 minutes of incubation, samples were centrifuged at 15.000 rpm for 15 minutes at room temperature. Supernatants were gently recovered and mixed with gasoline or diesel fuels. The emulsification index (EI24) has been determined as discribed in the afore section 2.2.

### 2.6. Extraction of Biosurfactant-like Molecule

Three methods have been used to extract the biosurfactant.

#### 2.6.1. HCL and Ethanol Precipitation

An overnight culture has been centrifuged at 13,000 ×g for 15 minutes. Once the supernatant was collected, HCl 1N and 90-degree ethanol were added to the supernatant. Precipitates have been generated by incubating samples at 4°C in overnight. Mixtures were centrifuged at 13000 g for 15 minutes to obtain granules. The granules obtained were tested with EI24 to evaluate the ability to emulsify the hydrocarbons.

#### 2.6.2. Ammonium Sulfate Precipitation Test

An overnight culture has been centrifuged at 13,000 rpm for 15 minutes to separate supernatant and pellet. Then, 15 mL of supernatant were mixed with ammonium sulfate (80%) for 15 minutes. Finally, this has been incubated with shaking overnight. The mixture has been centrifuged at 6000 rpm for 30 minutes at room temperature. Pellets were homogenized using PBS buffer. The emulsification activity has been assessed.

#### 2.6.3. Biosurfactant Extraction Using Chloroform

The 24 h culture was strictly centrifuged at 15,000*g* for 15 minutes to avoid any residual bacteria. One volume of supernatant was added with an equal volume of chloroform (v/v). The mixture was strongly agitated by a vortex. After centrifugation at 6000 rpm for 10 min, the nonaqueous phase is recovered. The solvent was allowed to evaporate completely only without heating above 40°C. The residue is dissolved in a PBS buffer. The emulsification activity is tested by mixing with gasoline or diesel fuel in comparison with the supernatant at the start point. The emulsification Index (EI24) has been determined.

### 2.7. Effect of Benzoic Acid and Salicylic Acid on Biosurfactant Secretion

Viability of *Shigella* strains has been first evaluated with different concentrations of benzoic acid and salicylic acid. *S. flexneri*5a M90T was grown in Luria-Bertani broth (LB) in the presence of various concentrations of benzoic acid (50 mM, 100 mM, 250 mM, and 500 mM) and salicylic acid (1.5 mg/mL, 3 mg/mL, 6.25 mg/mL, and 12.5 mg/mL). After that, all *Shigella* strains were grown in Luria-Bertani broth (LB) added with an adequate concentration of benzoic acid or salicylic acid at 37°C, during 24 hours. All supernatants were centrifuged and the secretion of biosurfactants was assessed by using emulsification assay (EI24).

### 2.8. Statistical Analysis

GraphPad Prism 7 and Excel software were used for analysis. The data represent the arithmetical averages of at least three replicates. Data were expressed as mean ± SD, and Student's test was used to determine statistical differences between strains and *p* < 0.05 was considered as significant. Principal component analysis (PCA) was used to investigate possible correlations between *Shigella* and emulsification index (EI24). Prior to ordination, percentage of emulsification activities data were transformed to better meet the assumptions of normality using ln (*x* + 1). All analysis was performed using CANOCO (Canonical Community Ordination, version 4.5).

## 3. Results

### 3.1. Screening for Biosurfactant Production

**Figure 1:**
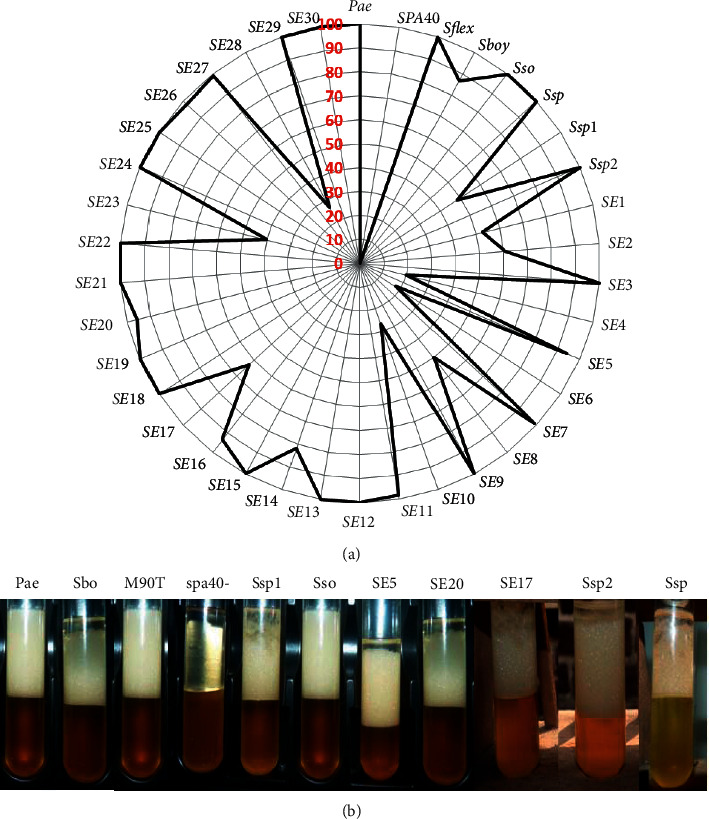
(a) Emulsification index percentage of all *Shigella* strains used in this study after 24 hours. Pae: *P. aeruginosa* used as positive control; M90T: *Shigella flexneri* 5a strain M90T; *spa40*-: *S. flexneri* spa40 mutant; Sbo: *S. boydii*; *Sso*: *S. sonnei*; Ssp, Ssp1, Ssp2: *Shigella* sp. from clinical strains; SE1 to SE30: *Shigella* sp. from environmental strains. (b) Emulsification index appearance of some *Shigella* strains.

In order to carry out our research, we first assess if *Shigella* strains would be able to produce BLM in extracellular medium.  Figure 1 shows that environmental strains and clinical strains are able to secrete BLM by showing emulsification percentages ranging between 15 % and 100 % (Figure 1(a)). *S. flex*neri spa40 mutant was not able to produce BLM compared with *Pseudomonas aeruginosa* used as positive control. The way of strains to produce BLM is shown in Figure 1(b). All strains are not represented (Figure 1(b)).

**Figure 2:**
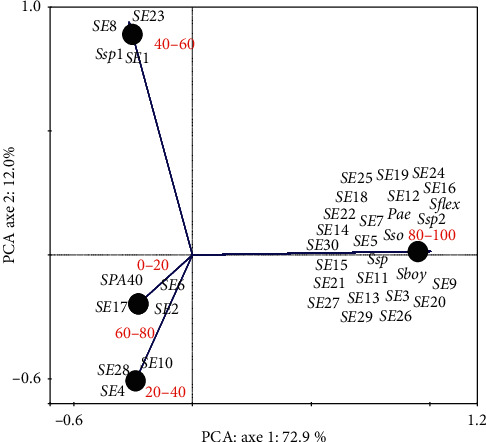
PCA of *Shigella* strains based on emulsification index (EI24). Pae: *P. aeruginosa* used as positive control; S. flex: *S. flexneri* M90T; S. flex-: *S. flexneri spa40* mutant; Sboy: *S. boydii*; Sso: *S. sonnei*; Ssp 1, 2: *Shigella* sp. from clinical strains; SE1 to SE30: *Shigella* sp. from environmental strains.

EI24 of some strains ranges from 80% to 100%. These included *Shigella flexeneri* M90T, *Shigella boydii* (Sbo), *Shigella sonnei* (Sso), *Shigella* sp (Ssp), Ssp2, SE3, SE5, SE9, SE11, SE12, SE13, SE14, SE15, SE16, SE18, SE20, SE21, SE22, SEI24, SE25, SE2626, SE27, SE29, and SE30 (Figure 2). The positive control has been found in this rate. SE1, SE8, SE23, and Ssp1 are ranging between 40% and 60%. SE4, SE10, and SE28 are between 20% and 40%. SE17 and SE2 from 60% to 80% and *Shigella* flexneri spa40 mutant (spa40-) are not able to produce biosurfactant, and SE6 is about 17% ranging from 0% to 20% (Figure 2).

### 3.2. Ability of *Shigella* Strain in the Swarming Test

**Figure 3:**
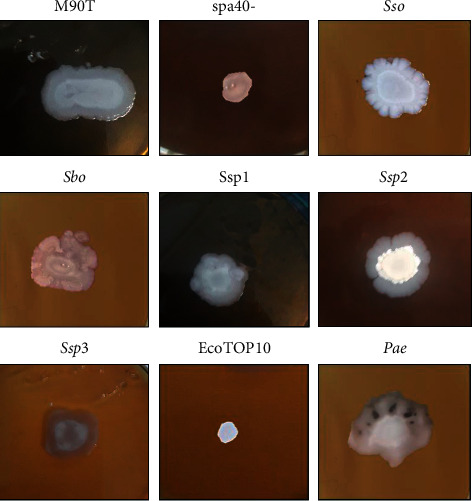
Swarming profile of *Shigella strains.* M90T: *Shigella flexneri* 5a strain M90T; Sbo: *S. boydii*; Sso: *S. sonnei*; *spa40-*: *S. flexneri* 5a *spa40-*; Ssp1, 2, 3: *Shigella* sp. Pae: *P. aeruginosa* used as positive control and *E. coli*-Top10 used as negative control.

Swarming is induced by the production of BLM. In order to demonstrate how *Shigella* could disseminate into epithelial cell, we first investigated if all *Shigella* strains used in this study were able to swarm by using 0.5% LB medium + 0.5% dextrose. As a result, *S. flexneri*5a M90T, *S. sonnei*, and *S. boydii* were able to spread and swarm. *spa40-* was not able to swarm (Figure 3). Some examples of the swarming profile of some *Shigella* strains after 24 hours are illustrated. We found that *S. flexneri spa40*- cannot swarm and *S. sonnei* have a particular swarming profile than other *Shigella* strains used in this study.

### 3.3. Bacterial Adhesion to Hydrocarbon (BATH)

**Figure 4:**
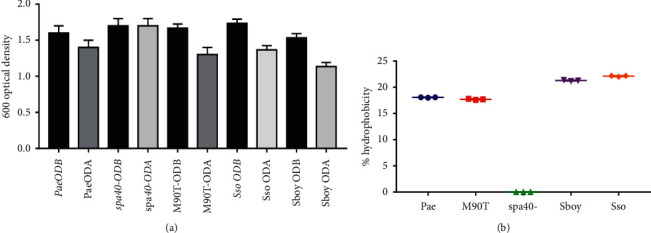
(a) *Shigella*'s adhesion to hydrocarbon phase of some strains used in this study. ODB: optical density before vortexing; ODA: optical density after vortexing; M90T: *Shigella flexneri* 5a strain M90T; Sbo: *S. boydii*; Sso: *S. sonnei*; spa40-: *S. flexneri* 5a spa40-; Pae: *P. aeruginosa* used as positive control. (b) Percentage hydrophobicity of *Shigella* strains.

To highlight the production of BLM by *Shigella* strains to induce interaction with hydrophobic areas, we performed analysis by evaluation of the ability to interact with hydrocarbon.  Figure 4 shows the bacteria adhesion profile of some *Shigella* strains. Only *S. flexneri* spa40 mutant does not interact with hydrocarbon area (Figure 4(a)). *S. flexneri*, *S. boydii*, and *S. sonnei* are positive with BATH techniques including a percentage of hydrophobicity of 17.64%, 21.42%, and 22.22%, respectively (Figure 4(b)).

### 3.4. Screening of Biosurfactant Secreted by *Shigella* sp

**Figure 5:**
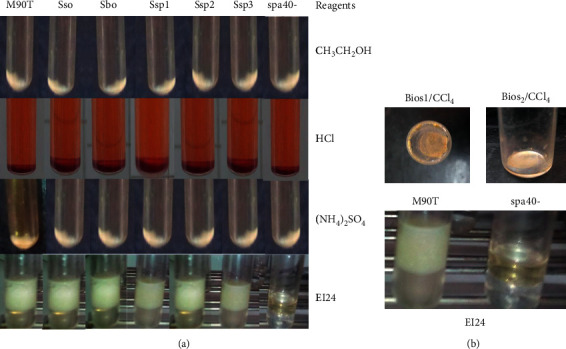
BLM purified from *Shigella* sp. TOP: profile obtained after precipitation with ethanol (CH_3_CH_2_OH), hydrochloric acid (HCl), and ammonium sulfate ((NH_4_)_2_SO_4_). EI24: emulsification index for all strains. *S. flexneri* M90T, *S. sonnei*, *S. boydii*, and *Shigella* sp.: Ssp1, 2, and 3. Bottom panel: residues obtained after evaporation of chloroform (CCl_4_) (left); emulsification index (EI24) for the extractable biosurfactant-like molecule (right). Bios1 and Bios2: biosurfactant-like molecule residues.

To highlight the biochemical specifications of the BLM secreted by *Shigella* strains used in this study, cultures of *Shigella* strains with supernatant-emulsified hydrocarbons (gasoline or diesel fuel) have been used to identify the type of biosurfactant-like molecules. Precipitation on hydrochloric acid, ammonium sulphate, and ethanol has been done. All strains showed a precipitate at the bottom of the tube (Figure 5(a)). The emulsification index after precipitation has been carried on EI24. Only the precipitate profile of the *S. flexneri spa40-* supernatant did not emulsify the gas oil and/or diesel fuel. *S*. *flexneri, S*. *sonnei, S*. boydii, and three *Shigella* sp. have 100% of EI24 (Figure 5(b)).

Strains with known hydrocarbon emulsification ability were selected from an organic solvent like chloroform using biosurfactant extraction assay. Biosurfactant could be extracted after evaporation of chloroform at 40°C from *S. flexneri* M90T. Nothing was obtained from *spa40*-. The extract after evaporation, suspended in PBS, was able to emulsify gasoline or gas oil with 100% of EI24 (Figure 5(b)).

### 3.5. BLM Is Secreted by Type Three Secretion System (T3SS)

**Figure 6:**
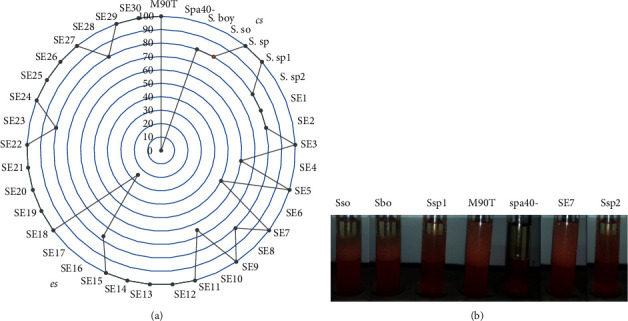
Emulsification index after Congo red induction. (a) cs: clinical strains; es: environmental strains; Pae: *P. aeruginosa* used as positive control; M90T: *Shigella flexneri* 5a strain M90T; *spa40*-: *S. flexneri spa40* mutant; Sbo: *S. boydii*; Sso: *S. sonnei*; Ssp, Ssp1, Ssp2: *Shigella* sp. from clinical strains; SE1 to SE30: *Shigella* sp. from environmental strains. (b) Emulsification index appearance of some *Shigella* strains.

Clinical strains including *S. flexneri*5a M90T, *S. sonnei*, *S. boydii*, three *Shigella* sp., and 30 environmental strains including SE1 to SE30 were cultivated to induce the secretion of effector on Congo red induction. *Shigella* species have been found to secrete BLM on Congo red induction conditions with EI24 ranging from 80% to 100%. The mutant *S. flexneri* spa40- did not emulsify the gasoline and/or diesel fuel in the presence of Congo induction with 0% of EI24 (Figure 6(a)). Emulsification index after Congo red type 3 secretion system of *Shigella* strain appearance are illustrated in Figure 6(b).

### 3.6. Effect of Benzoic Acid and Salicylic Acid on Biosurfactant Production

All *Shigella* strains were grown in Luria-Bertani (LB) broth in the presence of random concentrations of benzoic acid and salicylic acid (data not shown). We examined growth at the various concentration of benzoic acid including 50 mM, 100 mM, 250 mM, and 500 mM. As far as salicylic acid is concerned, 1.5 mg/mL, 3 mg/mL, 6.25 mg/mL, and 12.5 mg/mL were randomly selected. All *Shigella* strains grew normally within the physiological range of benzoic acid as determined by CFU per milliliter, but growth was significantly interesting at 100 mM benzoic acid and 1.5 mg/mL for salicylic acid (data not shown).

**Figure 7:**
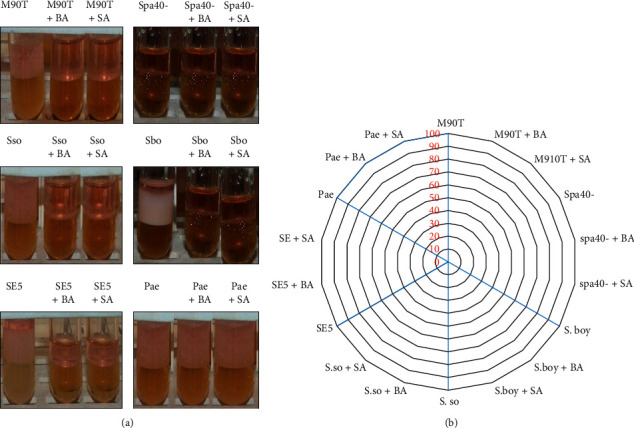
(a) Gasoline emulsifying activity of some *Shigella* strains used in this study with and without benzoic acid (BA) and salicylic acid (SA). M90T: *S. flexneri* strain M90T. spa40-: *S. flexneri spa40* mutant, Sso: *S. sonnei*; Sbo: *S. boydii*; SE: *Shigella* sp. (environmental strain); Pae: *P. aeruginosa.* (b) Gasoline emulsifying activity appearances of some *Shigella* strains tested with and without benzoic acid or salicylic acid.

To highlight the role of T3SS on the secretion of BLM, we assessed the effect of benzoic and salicylic acids to inhibit the biosurfactant production. Bacteria were previously incubated with 100 mM benzoic acid (BA) and 1.5 mg/ml salicylic acid (SA), and we showed that *S. flexneri* M90T, *S*. *sonnei*, *S*. *boydii*, and SE5 were not able to produce BLM with an emulsification index 0% EI24 (Figure 7(a)). This easily showed that all *Shigella* strains do not emulsify anymore gasoline or diesel fuel with benzoic acid or salicylic acid (Figure 7(a)). Strains are able to emulsify gasoline or diesel fuel without benzoic acid or salicylic acid. The appearance is also illustrated (Figure 7(b)). *P. aeruginosa* has been used as positive control since T3SS is widely conserved in most Gram-negative bacteria, and surprisingly *P. aeruginosa* was able to produce 100 % BLM in the presence or in the absence of BA and SA. It is worth noting that *spa40*- was not able to produce BLM neither in the presence nor in the absence of BA and SA as previously mentioned (Figure 7(a)7(a)).

## 4. Discussion

This work was conducted with the prime aim of contributing to the understanding of the *S. flexneri* 5a M90T epithelial cell invasion mechanisms. *Shigella* strains had been collected from the environmental areas, hospital, or laboratory. All strains had the ability to produce BLM during growth in extracellular medium, and the production is strictly depending on T3SS pathway. This result shows very clearly that these molecules are secreted in the extracellular medium as described by Usman et al. [10]. *spa40* mutant which has no T3SS, cannot secrete BLM. Several studies have demonstrated the role of T3SS in the secretion of numbers of effector proteins involved in invasion and dissemination [11, 12].

The emulsification index is a direct method for demonstrating the ability of strains to produce biosurfactants or not [5]. Those molecules have been known to form emulsions between two immiscible liquids [13, 14]. Experiments carried out from the acellular supernatant showed that *S. flexneri* 5a M90T as well as *S. boydii*, *S. sonnei,* and other *Shigella* sp. are able to emulsify gasoline and diesel fuels with EI24 ranging from 80% to 100%. Gram-negative bacteria are well documented to overcome this phenomenon. These include *P. aeruginosa* [13, 14], *Salmonella enteridis* [15], *Acinetobacter* sp. [16], and *Serratia Marcescens* [17]. Gram-positive bacteria are known as being efficient in producing BLM. The spore-forming bacteria like *B. subtilis*, *B. lichenifornis*, and *Lysinibacillus louembei* have been widely used to produce BLM [18–20].

Biosurfactants are native of several multicellular phenomena such as swarming described in several bacterial species [21]. By using specific culture media, we have shown that all strains of *Shigella* genus were positive in the swarming assay. The swarming phenomenon promotes the ability of biosurfactant production. This phenomenon is associated with antibiotic resistance, virulence, and biofilm formation in *Proteus mirabilis*, *Salmonella enterica* serovar Typhimurium, and *Serratia* [22–24]. This idea reinforces the fact that *Shigella* sp. could also use biosurfactants in its pathogenicity. No genes have been identified to be directly involved in BLM biosynthesis. In this work, we found that *ygaG* is a chromosomal gene of *S. flexneri* M90T. YgaG, which is the product of this gene, shares 90% of identity with LuxS involving in quorum sensing and biofilm formation [25, 26]. RhlA, RhlB, and RhlR proteins are known to promote the rhamnolipid secretion [27]. The secretion of biosurfactant is correlated with quorum sensing [28].

Pathogenicity in genus *Shigella* is determined by T3SS that has the ability to secrete a myriad of effector proteins into the target cells [29, 30]. In the absence of cellular contact, the secretory apparatus is not functional [31]; however, some proteins are secreted in leak condition. Cell contact is mimicked using Congo red [11]. Under the Congo red induction condition, all *Shigella* strains emulsified gasoline and diesel fuels, while the *S. flexneri* 5a M90T spa40 mutant did not emulsify them anymore. The mutant *S. flexneri spa40*- has no T3SS [11]. The *S. flexneri spa40*- in a noninducible condition [32] or in a Congo red induction condition does not produce BLM. In addition, by blocking T3SS using benzoic and salicylic acids compounds, we have demonstrated that BLM could not be secreted in extracellular medium. This confirms that BLM is secreted via T3SS. *P. aeruginosa* could secrete BLM in the presence or in the absence of inhibitors. This allows us to postulate that rhamnolipid molecule could use another pathway. An efflux mechanism is the top in *P. aeruginosa* [10, 33]. This inhibition assay with benzoic acid and salicylic acids showed a perfect correlation between the secretion of the BLM synthesized by *Shigella* and the inactivation of the type III secretion apparatus.

Regarding the BLM characteristics, precipitation assay such as hydrochloric acid, ammonium sulfate and ethanol allowed postulating that the secreted BLM could have a lipopeptide or peptide features. Only peptide or lipopeptide biosurfactants can precipitate at a very low pH or with ammonium sulphate [34, 35]. In proteomics studies, the sequential precipitation of ammonium sulfate proteins allows the proteins to be separated by “salting-in” or “salting-out” effect [36], which necessarily leads to the formation of protein aggregates and therefore to their precipitation. The BLM precipitate was able to emulsify gasoline and diesel fuel. Biosurfactants, like rhamnolipid, surfactin, and emulsan, are extractable by organic solvents [14, 37]. In addition, our study showed that the biosurfactant excreted by *Shigella* sp. is extractable with chloroform with higher efficiency and stability at 40°C.

BML are known to play several vital roles especially in the microbe's adhesion, bioavailability, desorption, and defense strategy. The most important role of microbial BLM is well reviewed for adhesion of the interfaces in cells–cells interactions [38]. *P. aeruginosa* is the best example of cell surface hydrophobicity which is justified by the presence of cell-bound rhamnolipid [39]. Our new finding showed that, by secreting BLM, *Shigella* sp. can easily bind to the cell hydrophobic interfaces by interacting with lipid rafts [30, 40–42]. By binding on cell membrane, BLM allows the reduction of the membrane tension and to help the translocon-like IpaB-C [43, 44] and the tip component IpaD [45, 46] to be close to the host membrane and automatically inserted inside the cytoplasmic membrane.

Many mechanisms have demonstrated how *S. flexneri* can disseminate inside epithelial cells [47, 48], helping to escape autophagy phenomenon [49] and to spread inside host cell [50] by using a specific domain of IcsB that interacts with cholesterol [30]. In this work, we showed that *S. flexneri*, *S. boydii,* and *S. sonnei* could spread using the swarming phenomenon. No studies have previously documented the ability of swarming in the mentioned conditions. This efficiently emphasized and amplified the idea that *Shigella* could be able to use several mechanisms that help spreading from cell to cell by secreting BLM. We are investigating the secretion of BLM inside epithelial cells. Based on our finding, we can propose that *Shigella* can invade and disseminate inside the epithelial cells using BLM pathways.

## 5. Conclusion

In order to contribute to the understanding of the mechanism of invasion of epithelial cells by *Shigella* sp., we have first shown that all *Shigella* strains as well as clinical or environmental strains are able to secrete biosurfactant-like molecules directly in the extracellular medium. Second, we have shown that the secretion of biosurfactants-like molecule depends on type three secretion system (T3SS). Our study suggests that the biosurfactant with lipopeptide or peptide features, stable at 40°C, could play an outstanding role in *Shigella* pathogenicity mechanisms including bacteria–host cell interaction, cell metabolism, and cell dissemination. This work contributes to the understanding of genes associated with a couple of components that are able to promote the biosynthesis, regulation, and secretion of BLM. Knocking out Shigella with couple genes encoded effector proteins such as IpaB, IpaC, and IpaD could orient investigation. In the continuation of our study experiments including MALDI-TOF and HPLC are in its ways to more biochemically characterize BLM. .

## Data Availability

The Excel sheets including the data used to support the findings of this study are available from the corresponding author upon request.
